# Spatiotemporal analysis of pulmonary tuberculosis in the central region of the Zhejiang Province, China (2016–2024)

**DOI:** 10.3389/fpubh.2025.1599274

**Published:** 2025-06-17

**Authors:** Kaixuan Zhang, Zuokai Yang, Jiamei Sun, Kui Liu, Qiaoling Lu

**Affiliations:** ^1^Department of Tuberculosis Control and Prevention, Shaoxing City Center for Disease Control and Prevention, Shaoxing, China; ^2^Department of Public Health Surveillance and Advisory, Zhejiang Provincial Science and Technology Program for Disease Prevention and Control (2025JK298), Hangzhou, China

**Keywords:** pulmonary tuberculosis, *Mycobacterium tuberculosis*, epidemiological characteristics, spatial autocorrelation, spatiotemporal clustering

## Abstract

**Background:**

In recent years, Shaoxing City, located in central Zhejiang Province, has experienced a slow decline in the incidence of pulmonary tuberculosis (PTB). Therefore, there is an urgent need to elucidate the potential causes for this decline through spatiotemporal analyses to provide a scientific basis for targeted prevention and control. We aimed to explore the spatiotemporal distribution of PTB notification rates in Shaoxing City from 2016 to 2024 and identify high-incidence clusters, thereby offering data-driven insights to optimize regional PTB control strategies.

**Methods:**

Statistical analyses were conducted using R and Excel on all reported active PTB cases in Shaoxing City. Spatiotemporal analysis of case distribution and regional clustering was conducted using ArcGIS and SatScan.

**Results and discussion:**

In total, 17,298 active PTB cases were registered between 2016 and 2024, including 9,749 laboratory-confirmed and 7,549 clinically diagnosed cases. The male-to-female ratio was 2.34:1. Farmers represented 68.2% of all cases. The PTB notification incidence showed a gradual decline. Spatial autocorrelation results revealed 52 sub-districts with high-high clusters over the nine-year period, primarily in Shengzhou and Xinchang counties. Spatiotemporal scan analysis identified one primary cluster area (*RR* = 1.62, *LLR* = 170.87, *p* < 0.001) and two secondary clusters between 2016 and 2024. The incidence of PTB in Shaoxing City showed a downward trend, though the decline was relatively slow. The southeastern region should be prioritized in efforts to accelerate the End TB Strategy. Overall, comprehensive and intensive interventions, such as large-scale chest X-ray screening and health education programs, should be enhanced to effectively curb PTB transmission, especially among males and farmers.

## 1 Introduction

Tuberculosis (TB) is a chronic infectious disease caused by *Mycobacterium tuberculosis* (*MTB*), primarily affecting the lungs (1). This places a heavy disease burden on low- and middle-income countries (2–4). Especially after the global pandemic of COVID-19, TB reemerged in 2023 as the leading cause of death from a single infectious pathogen (5). The number of deaths globally was approximately twice that of HIV/AIDS deaths during the same period, reaching nearly 1.25 million. According to the latest notification from the World Health Organization (WHO) (5), a total of 10.8 million new TB cases will be reported globally in 2023, with an incidence rate of 134 per 100,000 population. Of the estimated new cases, 55% were adult males, 33% were adult females, and 12% were children or adolescents. Thirty high-burden TB countries accounted for 87% of the total number of cases. China ranked third at 6.8%, with an estimated number of new tuberculosis cases of 741,000, an incidence rate of 52 per 100,000 population, 25,000 deaths, and a mortality rate of 2 per 100,000 population. Pulmonary tuberculosis (PTB), which accounts for more than 90% of tuberculosis cases, has consistently ranked second among all notifiable infectious diseases in China (6). Controlling the spread of TB has become a core public health issue in China and has attracted significant public attention.

Spatiotemporal analysis is widely used to study infectious diseases (7, 8). It was mainly used to reveal the distribution characteristics, temporal evolution laws, transmission routes, and potential risk areas of infectious diseases in geographical space, which would help to achieve early warning and intervention in epidemic situations. For example, during influenza seasons, spatiotemporal analysis can monitor the transmission of influenza viruses in real-time, including areas with a more severe epidemic situation and the speed of epidemic spread, providing timely information support for epidemic prevention and control (9). For the prevention and control of African swine fever (10), spatiotemporal analysis can identify high-risk areas and possible transmission routes, providing a scientific basis for epidemic prevention and control. Many domestic and foreign studies have shown that tuberculosis had a highly complex, dynamic, and spatially heterogeneous nature (11–15). In 2024, the PTB incidence rate in Shaoxing City was 30.58 per 100,000 people (unpublished data). Against the backdrop of the World Health Organization’s call to end TB, some actions such as launching tuberculosis-free communities and large-scale chest X-ray screening programs were performed in Shaoxing City. However, the decline in epidemic control has remained slow, highlighting the need for detailed exploration at finer spatial scales.

This study is the first to use a community or subdistrict-level units within Shaoxing City in Zhejiang Province. Unlike other studies that relied on counties as the unit of analysis, this approach employed a finer granularity and scale. This allowed for a deeper exploration of the potential risks associated with PTB occurrence, which enabled a more precise identification of high-risk areas. Furthermore, this study provides a robust scientific basis for targeted prevention and control interventions. The findings of this study also offer valuable new insights into regional policymaking and the implementation of differentiated prevention and control strategies.

## 2 Methods

### 2.1 Location

Shaoxing City is located in the north-central part of the Zhejiang Province, China, along the southern bank of Hangzhou Bay. Its geographical location ranges from 29°13′35″ to 30°17′57″ north latitude, and from 119°53′03″ to 121°13′37″ east longitude. Shaoxing City comprises six counties and districts: Yuecheng District, Keqiao District, Shangyu District, Xinchang County, Zhuji City, and Shengzhou City. It includes 105 subdistricts and townships. The average annual rainfall in Shaoxing City is approximately 1449.9 millimeters. Shaoxing City has a subtropical monsoon climate, with an annual average temperature of approximately 16–17°C. As of the end of 2024, the permanent resident population was 5.394 million.

### 2.2 Data collection

The details of all PTB cases were collected from the Tuberculosis Information Management System (TBIMS), which is a subsystem of the China Information System for Disease Control and Prevention. All active PTB cases diagnosed from 2016 to 2024, including both confirmed and clinically diagnosed cases, were included (16). Population data for the subdistricts were obtained from the official website of the Shaoxing Municipal Bureau of Statistics. For sub-districts that had undergone mergers or splits in the past, the two areas were treated as one unit when counting their risk. The Shaoxing vector map was sourced from the Shaoxing Public Service Platform for Geographic Information (17).

### 2.3 Definitions

Notified PTB cases comprised both laboratory-confirmed and clinically diagnosed patients. All cases were classified according to the National Diagnostic Criteria for Pulmonary Tuberculosis (WS 288–2008, WS 288–2017) and Classification of Tuberculosis (WS 196–2017) (16). Confirmed PTB cases were defined as those diagnosed based on etiological evidence, specifically including a positive sputum smear or culture or a positive result from a rapid molecular diagnostic test (e.g., GeneXpert), and meeting the clinical and radiological criteria for PTB. Clinically diagnosed PTB cases were defined as those exhibiting significant abnormalities on chest radiography or CT scans, accompanied by negative laboratory test results, or the absence of relevant testing while demonstrating a positive response to anti-TB treatment (18).

### 2.4 Statistical analysis

General epidemiological characteristics were analyzed using R (version 4.3.2) and Excel. Continuous data were presented as mean ± standard deviation, and comparisons of rates were performed using the chi-square test for trend.

Spatial autocorrelation: spatial autocorrelation refers to the correlation of a variable with itself across different spatial locations, and is an indicator of the degree of aggregation among spatial units. The basic measure of spatial autocorrelation is the spatial autocorrelation coefficient, which can be divided into global and local autocorrelation. Moran’s I index (19) is a statistical measure for detecting spatial autocorrelation and has been used to identify the spatial autocorrelation of tuberculosis. Moran’s I is defined as follows:


$$I=\frac{n}{\sum_{i=1}^{n}\sum_{j=1}^{n}w_{\mathrm{ij}}}*\frac{\sum_{i=1}^{n}\sum_{j=1}^{n}w_{\mathrm{ij}}(X_{i}-\bar{X})(X_{j}-\bar{X})}{\sum_{i=1}^{n}{\left(X_{i}-\bar{X}\right)}^{2}}$$

where *n* was the number of counties, *X_i_* and *X_j_* were the indicators of autocorrelations from unit index *i* and *j*. *W_ij_* was the matrix of spatial weights. If unit *i* is adjoined to unit *j*, *W_ij_* = 1; otherwise, W*_ij_* = 0. The Moran’s I index ranged between −1 and 1. A positive Moran’s I index indicates a positive correlation, with higher values indicating a stronger correlation. Conversely, a negative Moran’s I index indicated a negative correlation, suggesting a dispersed distribution. When the value is zero, there is no spatial clustering, indicating that the data are randomly distributed (20, 21). Statistical significance was tested using the standardized Z-score in ArcGIS 10.8.

Spatiotemporal clustering analysis: the discrete Poisson model was used to identify the spatiotemporal clustering of PTB using SaTScan 10.0.2 software (22). Spatiotemporal clustering analysis was used to determine whether clustering occurred in the temporal and spatial distributions of a disease. It integrates spatial and temporal dimensions to identify disease clusters, including time periods and clustering regions. In this study, the SaTScan 10.0.2 software was used for spatiotemporal scanning. The maximum spatial window was set at 30% of the total population, the maximum temporal window was set at 50% of the study duration, and the minimum temporal clustering was set at 1 year.

### 2.5 Ethics statement

This study was approved by the Ethics Committee of the Shaoxing City Center for Disease Control and Prevention. Since it was based on data from monitoring systems, all personal information was kept confidential, as required. This study conformed to the requirements of the Declaration of Helsinki and China’s Law on the Prevention and Treatment of Infectious Diseases.

## 3 Results

### 3.1 General characteristics

**Figure 1:**
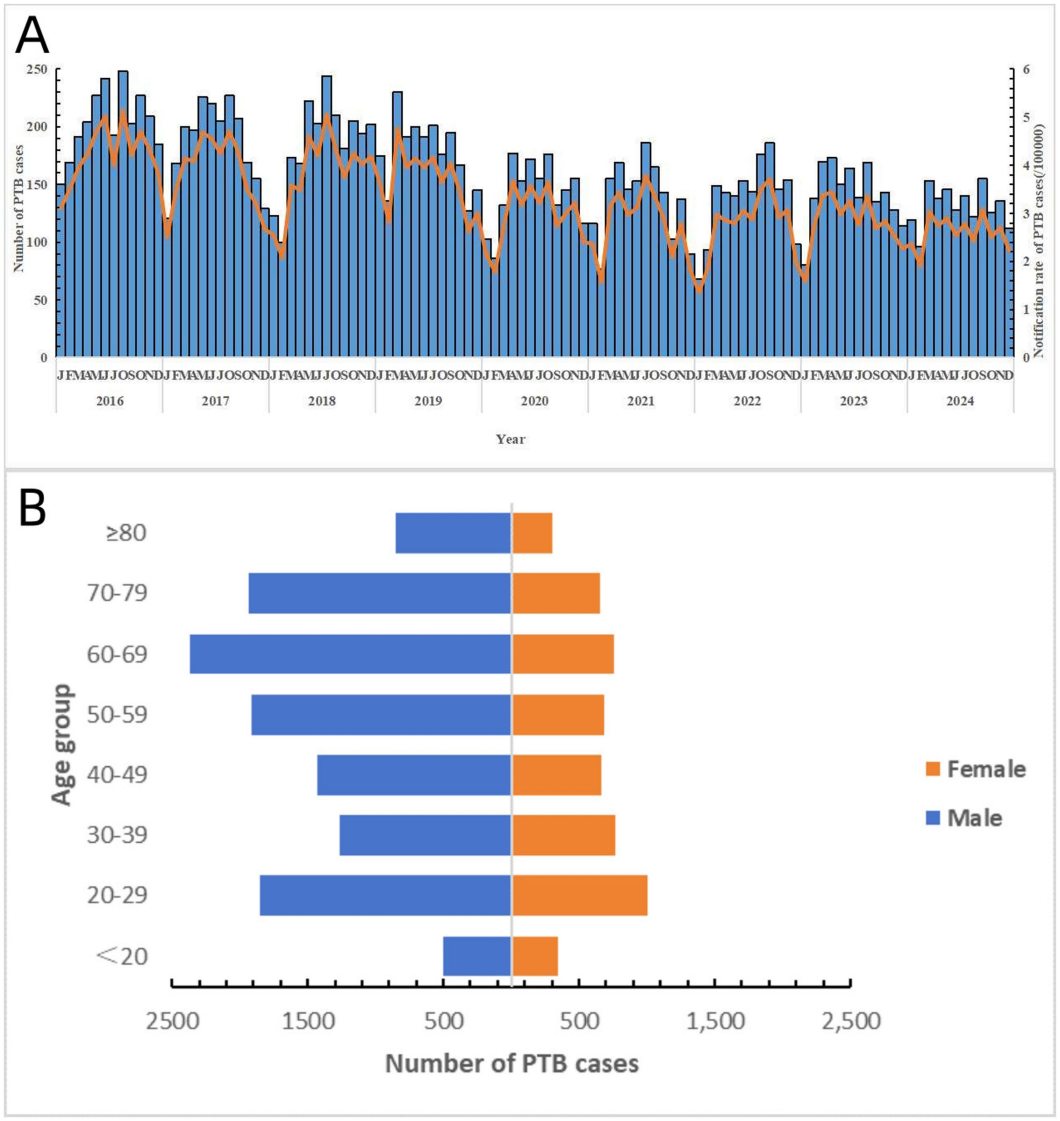
General epidemiological characteristics of PTB in Shaoxing City during the study period. **(A)** Notified case number and its rate of PTB in Shaoxing City by month. **(B)** Distribution of PTB cases by different sex and age group.

A total of 17,298 notified PTB patients were identified in Shaoxing City between 2016 and 2024. The average age was 50.85 ± 20.16 years, and the male-to-female ratio was 2.34:1. The average notification rate for PTB cases during the study period was 39.20 per 100,000 population. Specifically, the rate was 50.68 per 100,000 population in 2016, 47.10 per 100,000 population in 2017, 45.73 per 100,000 population in 2018, 43.84 per 100,000 population in 2019, 35.39 per 100,000 population in 2020, 32.99 per 100,000 population in 2021, 32.96 per 100,000 population in 2022, 33.83 per 100,000 population in 2023, and 31.21 per 100,000 population in 2024. The PTB notification rate showed an overall downward trend. The results of the trend chi-square test indicated a statistically significant difference in the notification rates of PTB from 2016 to 2024 (*χ*^2^ = 10.316, *p* = 0.001). The notification rate of PTB exhibited certain seasonal patterns, with peaks during spring and summer. In terms of the occupational distribution of all PTB cases, farmers accounted for the largest proportion (68.2%), followed by workers (12.5%), retirees (4.2%), students (4.1%), housework and unemployed (3.6%), and others (7.4%) (Figure 1).

### 3.2 Global spatial autocorrelation

The results of the global spatial autocorrelation analysis showed that the distribution of the notification rate of PTB patients in 2016–2020 and 2023–2024 was spatial heterogeneous, whereas it was not present in 2021 and 2022 (Table 1).

**Table 1:** Global spatial autocorrelation analysis of PTB notification rate from 2016 to 2024.

Year	Moran’s I index	Moran’s I Z-score	Moran’s I *P*-value
2016	0.157	2.785	0.005
2017	0.187	3.235	0.001
2018	0.167	2.912	0.004
2019	0.290	4.924	<0.001
2020	0.186	3.206	0.001
2021	0.102	1.852	0.063
2022	0.009	0.306	0.759
2023	0.113	2.007	0.045
2024	0.131	2.300	0.021

### 3.3 Local spatial autocorrelation

**Figure 2:**
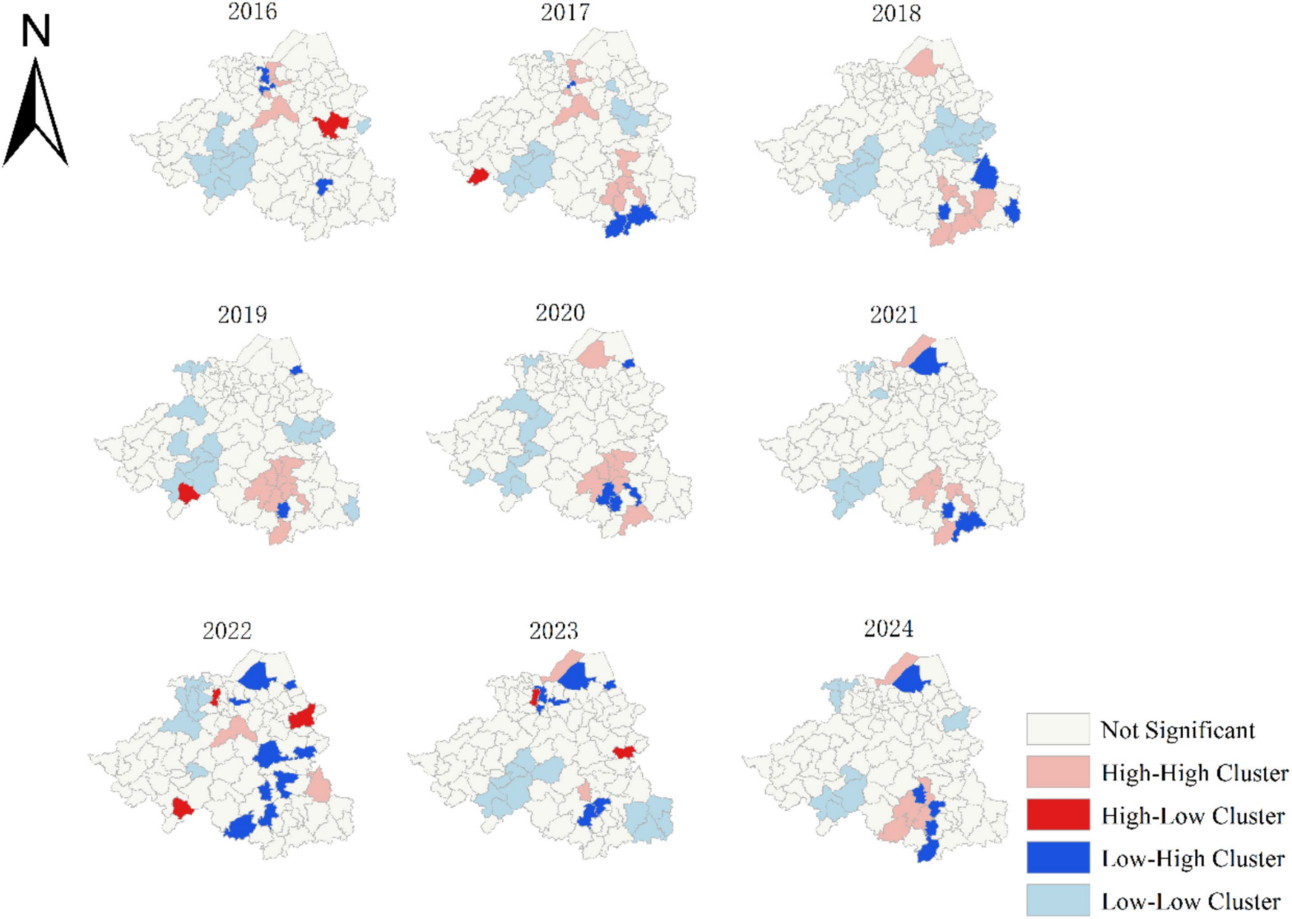
Local spatial autocorrelation analysis of PTB notification rate from 2016 to 2024.

The results of the local spatial autocorrelation analysis showed that there were 52 high-high clustering areas over the 9-year period. These high-high clustering areas gradually shifted from the central region of Shaoxing to the southeast in 2016. They temporarily disappeared between 2022 and 2023, and reappeared in the southeast by 2024. However, low-low clustering areas remained consistent in the southwestern region. Among the high-high clusters, the Qixing Sub-district had the highest number of occurrences (five), followed by the Ganlin (four), Lushan (four), Sanjiang (four), Chentan (three), Maan (three), Pukou (three), Nanming (three), and Shanhu sub-districts (three). All of them, except for the Qixing Sub-district, are under the jurisdiction of Shengzhou City (Figure 2).

### 3.4 Spatiotemporal clustering of PTB

A spatiotemporal scan analysis of the PTB notification rate in Shaoxing City from 2016 to 2024 identified significant clusters, including one primary cluster covering 19 subdistricts in Xinchang County and Shengzhou City from 2016 to 2019 (*RR* = 1.62, *LLR* = 170.87, *p* < 0.001), and two secondary clusters covering the northern part of Shaoxing City (2016–2019; *RR* = 1.39, *LLR* = 122.81, *p* < 0.001) and the western part of Shaoxing City (2016–2019; *RR* = 2.20, *LLR* = 122.52, *p* < 0.001). Additional details are summarized in Table 2.

**Table 2:** Spatiotemporal scan analysis of PTB notification rates.

Cluster type	Cluster area	Cluster time	Radius (km)	Observed/expected	RR	LLR	*P*
Primary cluster	Chengnan, Nanming, Yulin, Qixing, Dongming, Chengtan, Ruao, Huangze, Wozhou, Sanjiang, Jingling, Lushan, Qiaoying, Shanhu, Jinting, Pukou, Ganlin, Shaxi, Xiaojiang Subdistrict	2016–2019	22.47	1.55	1.62	170.87	<0.001
Secondary cluster (1)	Dongguan, Daoxu, Caoe, Baiguan, Changle, Songxia, Taoyan, Lianghu, Fusheng, Yiting, Sunduan, Shangpu, Xiaoyue, Xietang, Lihai, Gaibei, Tangpu, Mashan, Fenghui, Jishan, Donghu, Didang, Pingshui, Dingzhai, Fushan, Tashan, Doumen, Zhangzhen, Chengnan, Yonghe, Maan Subdistrict	2016–2019	23.96	1.32	1.39	122.81	<0.001
Secondary cluster (2)	Datang, Tangzhu Subdistrict	2016–2019	6.22	2.17	2.20	122.52	<0.001

## 4 Discussion

Most spatial analyses of tuberculosis (TB) have been conducted at the county or prefecture levels as the smallest research units (23, 24). A large spatial scale may lead to an overly generalized identification of risk factors, thereby reducing the accuracy of the models and their practical application. Moreover, during the study period, the administrative merging and splitting of certain counties complicated in-depth analyses of the same geographic area, an issue that could be significantly mitigated if the analyses were conducted at the subdistrict level. Shaoxing City was selected as the study site. Using the subdistrict as the smallest unit for PTB risk assessment, more precise identification of high-risk areas for PTB was performed, thus laying the foundation for future targeted TB interventions.

In this study, the PTB notification rate in Shaoxing City showed a declining trend from 2016 to 2024, with the lowest and highest rates recorded in 2024 and 2016, respectively. During this period, the promotion of rapid diagnostic technologies has significantly reduced the time required to diagnose PTB and drug-resistant PTB cases, offering higher sensitivity and specificity than traditional smear microscopy (25). Additionally, the reimbursement rate for medical insurance has increased to at least 70% for general PTB patients and at least 90% for drug-resistant PTB patients, effectively reducing the financial burden on these patients (26). These measures collectively contributed to the observed decline in the PTB notification rate over time. The slight fluctuation in the PTB notification rate in 2023 may be attributed to the COVID-19 pandemic. During the strict control stage (January 23, 2020, to May 31) and the regular control stage (June 1, 2020, to January 7), patients with mild symptoms were less willing to seek medical care, leading to delayed diagnosis of some PTB cases and an unusual decline in PTB notification rates (27, 28). Following the relaxation of COVID-19 restrictions in 2023, healthcare resources gradually recovered, and previously undiagnosed or untreated PTB cases began to reenter the healthcare system. Additionally, in 2023, the Zhejiang Province initiated the project of “construction of provincial-level TB-free communities.” This comprehensive implementation included expanding the scope of active PTB screening, particularly targeting individuals aged 65 years and above for chest radiography or additional latent infection screening for individuals at potential risk. These measures have further contributed to the decline of TB epidemics.

Demographically, PTB was predominantly found in males and farmers. This aligns with the global trend of a higher PTB incidence in males compared than in females (4). This sexual disparity may have resulted from a combination of genetic and environmental factors. Polymorphisms in immune-related genes (HLA, TLR, and VDR) may influence individual immune responses to *MTB*, leading to differences in the onset of infection between males and females (29, 30). Some evidence has shown that males possess only one X chromosome, potentially creating a disadvantage in the expression of certain immune-related genes, thereby increasing their susceptibility to infection (31). Environmental factors also play an indispensable role. Males often bear greater economic responsibilities and societal/family role pressures, which may lead them to neglect their health and increase their risk of PTB (32). Additionally, unhealthy habits such as smoking and excessive alcohol consumption, which are more prevalent among males, weaken immune defenses, further increasing PTB vulnerability (33). The farming population also exhibits a high PTB risk, likely linked to poor living conditions, rudimentary working environments, and inadequate healthcare access among this group (34). Furthermore, an interaction may exist between these two high-risk groups, especially among male farmers who face double risk, amplifying their PTB risk. Therefore, PTB prevention strategies should be considered for individuals who are more susceptible to PTB.

Further analysis revealed significant spatial heterogeneity in PTB epidemics during the study period. Local spatial autocorrelation analysis indicated that high-high clustering areas have gradually shifted southeastward since 2016. This spatial trend may be attributed to three clustered outbreaks in Xinchang County in 2017 and four additional clustered outbreaks in Shengzhou City. However, in 2024, these high-high clustering areas will re-emerge in the southeastern region, highlighting that Shengzhou City and Xinchang County should still focus on PTB control and prevention. Shengzhou City accounted for 38.4% of the 52 high clusters, while Xinchang County accounted for 34.6%. This may be attributed to the fact that both Shengzhou and Xinchang were typical mountainous areas within Shaoxing City. The complex terrain in these regions often delays the diagnosis and treatment of PTB, thus increasing the risk of infection and disease transmission (35, 36). Additionally, relatively underdeveloped socioeconomic conditions and limited healthcare resources in mountainous areas may facilitate the spread of PTB. To address these challenges, it is recommended that combined active PTB and latent infection screening be prioritized in Shengzhou and Xinchang Counties.

Based on health checkups for the older adult within the Basic Public Health Services project, referral tracking and further diagnosis of those with chest X-ray findings suggestive of PTB were performed. Meanwhile, by leveraging the “Tuberculosis-Free Community Project,” LTBI testing was conducted in the older adult population. In regions with adequate health resources, preventive treatments should be offered to individuals with positive LTBI results. Furthermore, in areas where conditions permit, pilot programs for subsidized inpatient isolation can be initiated to support the management of active cases. Through these combined efforts, we can enhance the detection of active pulmonary tuberculosis, reduce infections within communities and households, and decrease the reactivation of latent infections.

Based on the spatiotemporal analysis results, some sub-districts were identified as high-risk areas for PTB. The Qixing Subdistrict, as the core area of Xinchang County, it has the highest population density and significant population mobility, increasing the risk of *MTB* transmission (37). The Ganlin Subdistrict in Shengzhou City is primarily agricultural, with relatively low economic development. Some residents may face challenges, such as limited access to healthcare resources and insufficient health awareness (38), leading to lower rates of early PTB detection and treatment. The Lushan and Sanjiang sub-districts, located in the urban area of Shengzhou City, have a high degree of urbanization and a large migrant worker population. Further statistical analysis revealed that 29.74% of the PTB cases reported in Lushan and Sanjiang from 2016 to 2024 were non-local residents, compared with 24.63% of the entire patient population. Therefore, we speculated that the influx of migrant workers may be a significant factor contributing to the increased risk of PTB transmission in these areas. In the future, we plan to conduct health surveys and targeted screening for latent infections in factories and communities in these regions. This study is the first subdistrict-based research conducted in the central region of the Zhejiang Province utilizing spatiotemporal analysis methods to explore potential risk areas, thereby enabling more precise identification of high-risk zones.

However, there are some limitations. The study covered an extended period, during which limited townships and sub-districts experienced administrative reorganizations, mergers, and adjustments. To mitigate the potential impact, we standardized the data based on the 2024 administrative divisions of Shaoxing City. However, there was still a certain degree of impact. In addition, given the retrospective nature of the study, some potential asymptomatic cases may have been missed by passive case detection through TBIMS may not have been detected, which could cause bias.

## 5 Conclusion

In summary, the PTB epidemic in Shaoxing City declined from 2016 to 2024 at a relatively slow rate. The southeastern region of Shaoxing City has been identified as a priority area for the active screening of active PTB and latent infections. In the future, it will be essential to leverage various public health initiatives to enhance the early detection, diagnosis, and treatment of PTB, which could reduce the risk of TB transmission and contribute to more effective control of PTB.

## Data Availability

The raw data supporting the conclusions of this article will be made available by the authors, without undue reservation.
